# Joining of Ti6Al4V to Al_2_O_3_ Using Nanomultilayers

**DOI:** 10.3390/nano12040706

**Published:** 2022-02-21

**Authors:** Marcionilo Silva, Ana Sofia Ramos, Maria Teresa Vieira, Sónia Simões

**Affiliations:** 1 Department of Mechanical Engineering, Federal University of Amazonas, General Rodrigo Octavio Jordão Ramos ST., Manaus 69067-005, Brazil; marcionilo@ufam.edu.br; 2 Department of Metallurgical and Materials Engineering, University of Porto, Rua Dr. Roberto Frias, 4200-465 Porto, Portugal; 3 University of Coimbra, CEMMPRE, Department of Mechanical Engineering, R. Luís Reis Santos, 3030-788 Coimbra, Portugal; sofia.ramos@dem.uc.pt (A.S.R.); teresa.vieira@dem.uc.pt (M.T.V.); 4 LAETA/INEGI—Institute of Science and Innovation in Mechanical and Industrial Engineering, Rua. Dr. Roberto Frias, 4200-465 Porto, Portugal

**Keywords:** diffusion bonding, nanomultilayers, Ti6Al4V, Al_2_O_3_, sputtering, microstructure, interface

## Abstract

Diffusion bonding of Ti6Al4V to Al_2_O_3_ using Ni/Ti reactive nanomultilayers as interlayer material was investigated. For this purpose, Ni/Ti multilayer thin films with 12, 25, and 60 nm modulation periods (bilayer thickness) were deposited by d.c. magnetron sputtering onto the base materials’ surface. The joints were processed at 750 and 800 °C with a dwell time of 60 min and under a pressure of 5 MPa. Microstructural characterization of the interfaces was conducted by scanning electron microscopy (SEM) with energy-dispersive X-ray spectroscopy (EDS), and electron backscatter diffraction (EBSD). The mechanical characterization of the joints was performed by nanoindentation, and hardness and reduced Young’s modulus distribution maps were obtained across the interfaces. The joints processed at 800 °C using the three modulation periods were successful, showing the feasibility of using these nanolayered films to improve the diffusion bonding of dissimilar materials. Using modulation periods of 25 and 60 nm, it was also possible to reduce the bonding temperature to 750 °C and obtain a sound interface. The interfaces are mainly composed of NiTi and NiTi_2_ phases. The nanoindentation experiments revealed that the hardness and reduced Young’s modulus at the interfaces reflect the observed microstructure.

## 1. Introduction

The joining of metal to ceramics has attracted the attention of the scientific community due to the possibility of combining the properties of these dissimilar materials, providing unique advantages for new engineering applications, for instance, in heat-exchangers, thermoelectric, medical, semiconductors, and micro-electro-mechanical systems [1,2,3]. Titanium alloys are the most common when lightweight and strength are requested. For instance, the Ti6Al4V alloy has excellent mechanical properties, such as high creep resistance and high-temperature specific strength [4]. Furthermore, relating it with advanced ceramics, such as alumina (Al_2_O_3_), that has high thermal stability, low density, high wear resistance, and chemical inertness [5] can complement their advantages in terms of performance and economy [6]. The joining of Al_2_O_3_ to titanium alloys can promote the production of advanced components combining the advantages of the two materials and it can be used in vacuum pipes, energy converters, semiconductor devices, missiles, rockets and satellites. However, the bonding of these materials cannot be achieved using traditional fusion welding processes due to the differences in thermal conductivity, coefficient of thermal expansion (CTE), and chemical properties. The residual stress during cooling is the primary effect detrimental to the reliability of the joint [7]. Several technologies have been applied to join dissimilar materials [1]. However, the most reported metal to ceramic joining processes are brazing [8,9,10,11,12] and solid-state diffusion bonding [13,14,15,16,17,18].

Brazing allows successful joints to be obtained between metallic and ceramic base materials as it does not involve melting the base materials, thus reducing the formation of residual stresses [1,2,8,9,10,11,12]. Nevertheless, the most reported brazing alloys in the literature for brazing Al_2_O_3_ to Ti6Al4V are Ag-based alloys which, despite requiring a low processing temperature, promote the formation of (Ag), which compromises the service temperature of the joints [1].

Diffusion bonding [13,14,15,16,17,18] can be a more appropriate joining technology for dissimilar bonding between titanium alloys and advanced ceramics. However, the conventional diffusion bonding process involves high temperature, pressure, and dwell time which causes thermal stress due to a mismatch in coefficient of thermal expansion (CTE) and thermal conductivity; consequently, cracks come up at the joint’s interface, mainly during cooling [1]. Thus, several approaches have been developed to join dissimilar materials by diffusion bonding, like applying interlayers between the base materials to be joined [17,18,19,20,21,22,23,24]. The interlayer has a crucial role in reducing the temperature and pressure of the bonding process, and consequently, the thermal stress, besides increasing the contact area that favors achieving high strength joints. In the last years, dissimilar metals have been joined by diffusion bonding using as interlayers reactive multilayer thin films with nanometric bilayer thickness (modulation period (Λ)) [25,26,27]. The success of the multilayers’ application depends on the chemical elements that constitute them, for example, Ti/Al [25], Ni/Al [26] or Ni/Ti [27], whose exothermic reaction works as an additional localized heating source. For Ti/Al, Ni/Al and Ni/Ti multilayers, the heat released is ~434, ~562 and ~551 J/g, respectively [28]. This additional heat source allows sound joints to be obtained under less demanding conditions, without compromising the mechanical properties. Nevertheless, this approach has not been widely studied for joining metals to ceramics. Only a few reports are available in literature using alternating nanolayers to join metals to ceramics [20,29,30].

Zhu and Włosiński [29] deposited Ti films onto aluminum nitride (AlN) surface by RF sputtering and joined the coated AlN to Cu by diffusion bonding at 900 °C for 30 min under a pressure of 6 MPa. The shear tests showed a strength value of ~35 MPa. The element distribution across the interface shows intense diffusion of Cu and Al from the base materials and an interface rich in Ti. Moreover, the authors coated some Cu samples with Ni-Al films deposited by plasma spraying (~800 µm thick), increasing the shear strength up to ~60 MPa [29].

Cao et al. [30] investigated the joining between TiAl and TiC using an interface formed by Ni/Al multilayers with a total thickness of ~30 µm deposited by magnetron sputtering. The joints were processed by diffusion bonding at 700 °C, dwell time of 10 min under a pressure of 40 MPa. The microstructure revealed intense diffusion of Ti from the TiAl base metal to the interface. However, it was not identified Ti in the zone adjacent to TiC. The Ni diffusion occurred with less intensity from the interface towards the TiAl. The microstructural characterization showed that several layers formed at the interface; AlNi_3_, AlNi_3_ + AlNi, Ni_3_(AlTi), Ni_2_AlTi, (Ni, Al, Ti) phases.

Yi et al. [20] investigated the joining between copper and alumina by diffusion bonding using Ti/Al multilayers with different modulation periods and Al:Ti atomic ratios. The joints were processed at 900 °C for 15 min under 5 MPa. The results showed that sound joints could be obtained, and the shear tests revealed that the best result (~68 MPa) was achieved using higher periods (880 nm) and a total thickness of 38 µm [20].

Preliminary authors’ work [23] on the diffusion bonding of Al_2_O_3_ to Ti6Al4V using a reactive Ni/Ti multilayer with a 50 nm modulation period confirmed that this could be an interesting approach for joining these base materials. However, the applied pressure was too high, which caused a significant plastic deformation of the Ti6Al4V alloy. The decrease of pressure is a crucial aspect for implementing this approach.

In this context, the present work’s objective is to study the feasibility of joining Ti6Al4V to Al_2_O_3_ by diffusion bonding using Ni/Ti reactive multilayer thin films prepared by magnetron sputtering and reducing the pressure to avoid the plastic deformation of the Ti alloy. Moreover, the influence of different nanometric modulation periods (12, 25, and 60 nm) was studied. The bilayer thickness (period) of the multilayers is a critical parameter since it influences their reactivity and grain size. According to the work of Adams et al. [31], the peak reactivity of free-standing Ni/Ti multilayers is observed at ~10–15 nm, although a high reaction velocity is observed up to a ~60 nm bilayer thickness. The use of the nanostructured multilayers as an interlayer material aims at increasing the diffusivity at the interface, promoting the formation of an interface without chemical and structural discontinuities. The total thickness of these nanomultilayers is close to 2.5–3.0 µm. The bonding experiments were conducted at 750 and 800 °C under a pressure of 5 MPa. The microstructural characterization of the joint’s interface was performed by scanning electron microscopy (SEM) and energy-dispersive X-ray spectroscopy (EDS), and electron backscatter diffraction (EBSD), while the mechanical characterization was performed by nanoindentation across the joint’s interface.

## 2. Materials and Methods

### 2.1. Base Materials

Ti6Al4V alloy and Al_2_O_3_ were purchased from Goodfellow in rods with diameters of 7 and 6 mm, respectively. They were cut ~5 mm in length, ground carefully to obtain parallelism between the two surfaces of each substrate, followed by polishing down to 1 µm diamond suspension and 0.06 µm colloidal silica. The rods of the Al_2_O_3_ were encapsulated by cold mounting in epoxy resin before the cut to avoid cracks and surfaces damage. The base materials were cleaned with acetone, ethanol, and deionized water in an ultrasonic bath and dried with heat blow air before the depositions. After applying the metallographic procedure, the average surface roughness (Ra) was ~0.06 and ~0.10 µm for Ti6Al4V and Al_2_O_3_, respectively.

### 2.2. Deposition of Ni/Ti Nanomultilayer Thin Films

**Figure 1:**
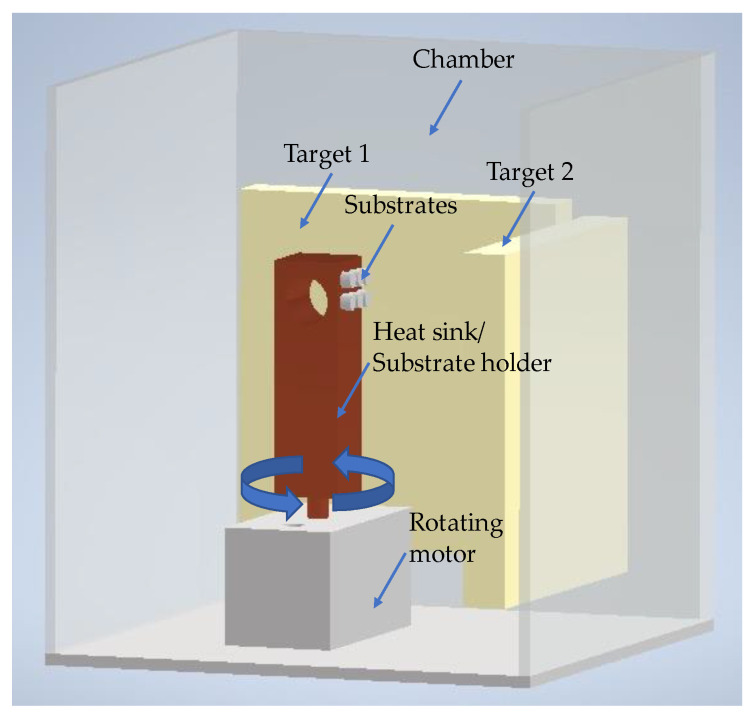
Scheme of the deposition chamber of the magnetron sputtering equipment used to prepare the Ni/Ti nanomultilayer thin films.

Nickel and Titanium nanolayers were alternately deposited onto the polished surfaces of both base materials (substrates) by direct current(d.c.) magnetron sputtering using titanium (99.99% pure) and nickel (Ni-7V wt.%) targets (150 mm × 150 mm × 3–7 mm thick). After achieving a base pressure below 5 × 10^−4^ Pa in the sputtering chamber, Ar was introduced (P~1.5 × 10^−1^ Pa), and the substrates were cleaned by heating followed by Ar+ (current of 20A) etching using an ion gun. The Ni/Ti depositions, carried out at a 4 × 10^−1^ Pa Ar pressure, started immediately after the substrates’ cleaning. To avoid excessive substrate heating and thus prevent reactions during the deposition process, the substrates were glued with a silver drop to a thick copper block substrate holder that acted as a heat sink. In addition, a silicon sheet was also glued to the copper block to obtain nanomultilayer thin films similar to those deposited onto the Ti alloy and alumina. The coated Si contributed to identifying the morphology and structure of the nanomultilayers and to obtaining the total thickness and average chemical composition of the nanolayered films. The total thickness was measured by profilometry (Perthometer SP4, with laser probe (Mahr Perthometer SP4, Göttingem, Germany)) on the Si substrates. Figure 1 shows a scheme of the deposition chamber.

The power density applied to the Ni and Ti targets was ~2.8 × 10^−2^ Wmm^−2^ and ~7.2 × 10^−2^ Wmm^−2^, respectively, in order to obtain a near equiatomic average chemical composition (50 at. % Ni: 50 at.% Ti). The Ni target has vanadium to eliminate its ferromagnetism [32]. As the third element in B2 -NiTi (cubic austenite phase), vanadium can be in both Ni and Ti substitutional sites [33]. The distance between the targets and substrates’ surface was 90 and 75 mm for Ni and Ti targets, respectively. The substrate holder’s rotation speed defines the time that the rotating substrates are in front of each target, determining the thickness of the individual layers, and consequently, the period or bilayer thickness. The Ni/Ti nanomultilayer thin films were produced with modulation periods (Λ) of 12, 25, and 60 nm and the rotation speed of the substrates was ~8, 3.3, and 2 rpm, respectively. These periods were selected based on the reactivity of Ni/Ti multilayers and on previous dissimilar joining of metallic materials by diffusion bonding [31,34]. A deposition time of ~30 min was selected for a total thickness close to 2.5–3.0 µm. Titanium’s bottom nanolayer ensured a good adhesion to the base materials [24]. Adhesion between Ni/Ti thin films and substrate is particularly relevant for the Al_2_O_3_ base material because the lack of adhesion can compromise the diffusion bonding process. The adhesive strength between nanomultilayer thin films and Al_2_O_3_ substrate was evaluated in a previous work [23]. The top nanolayer was Ni to prevent the Ti-O interaction and, thus, the oxidation of the nanomultilayers.

### 2.3. Diffusion Bonding Process

**Figure 2:**
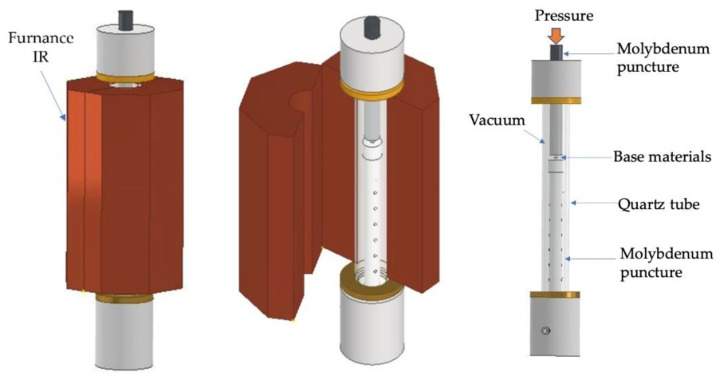
Schematic draw of the diffusion bonding apparatus.

The joints were performed in an apparatus consisting of a mechanical testing machine (LLOYD Instruments LR 30K (AMETEK Test & Calibration Instruments Lloyd Materials Testing, West Sussex, UK), vertical infrared radiation furnace (Termolab Electrical Furnace, Agueda, Portugal), molybdenum punctures to apply pressure, quartz tube, and vacuum system described in previous work [27]. A scheme of the diffusion bonding apparatus used in this work is presented in Figure 2. The diffusion bonding experiments were performed at a vacuum level better than 10^−2^ Pa using Ni/Ti reactive nanomultilayers with different modulation periods to evaluate their feasibility of enhancing joining Ti6Al4V to Al_2_O_3_. The diffusion bonding was carried out at 750 and 800 °C, by applying 5 MPa for 60 min. The heating rate was 10 °C.min^−1^ up to the bonding temperature, and the cooling rate was 5 °C·min^−1^ up to 500 °C, followed by 3 °C·min^−1^ down to room temperature.

### 2.4. Microstructural Characterization of the Nanomultilayers and Joints’ Interface

The microstructural and chemical characterization of the nanomultilayers and joints interface was carried out by scanning electron microscopy (SEM) (Thermo Fisher Scientific QUANTA 400 FEG SEM, Thermo Fisher Scientific, Hillsboro, OR, USA) operating at an accelerating voltage of 15 keV, coupled with energy-dispersive X-ray spectroscopy (EDS (EDAX Inc. (Ametek), Mahwah, NJ, USA)). The EDS measurements were made at an accelerating voltage of 15 keV by the standardless quantification method. The results obtained by this method provide a fast quantification with automatic background subtraction, matrix correction, and normalization to 100% for all the elements in the peak identification list. Monte Carlo simulations of electron trajectories using CASINO software estimated the volume of interaction. The estimated values for the lateral spread and depth of the interaction volume are, respectively, 1.5 and 1.4 μm for the Ti6Al4V and 1.2 and 0.8 μm for the NiTi and NiTi_2_. The cross-sections of the films and joint interfaces were prepared using standard metallographic procedures.

EBSD data were analyzed using TSL OIM Analysis 5.2 2007 (EDAX Inc. (Ametek), Mahwah, NJ, USA). To identify the crystallographic phases formed at the joint interface, analyses were conducted using an acceleration voltage of 15 keV to obtain Kikuchi patterns. The indexation of the patterns was made by ICDD PDF2 (2006) database.

### 2.5. Mechanical Characterization

Nanoindentation tests were carried out across the joints’ interfaces using Micro Materials-NanoTest equipment with a Berkovich diamond indenter (NanoTest, Micro Materials Limited, Wrexham, UK). The depth-sensing indentation tests were performed in load control mode, up to a maximum load of 5 mN. Indentation matrixes formed by 8 rows and 12 columns (96 measurements) were defined along the joint interface. Each matrix starts on the Ti6Al4V side, crosses the joint interface, and finishes on the alumina side. The distance between columns was 3 µm to guarantee that some indentations fall in the region corresponding to the interface, and the distance between rows was 5 µm. Fused quartz was used as reference material to determine the Berkovich tip area function. Hardness and reduced Young’s modulus were determined by the Oliver and Pharr method [35]. Before the indentation tests, the joints were polished using standard metallographic procedures.

## 3. Results and Discussion

### 3.1. Ni/Ti Nanomultilayers Characterization

**Figure 3:**
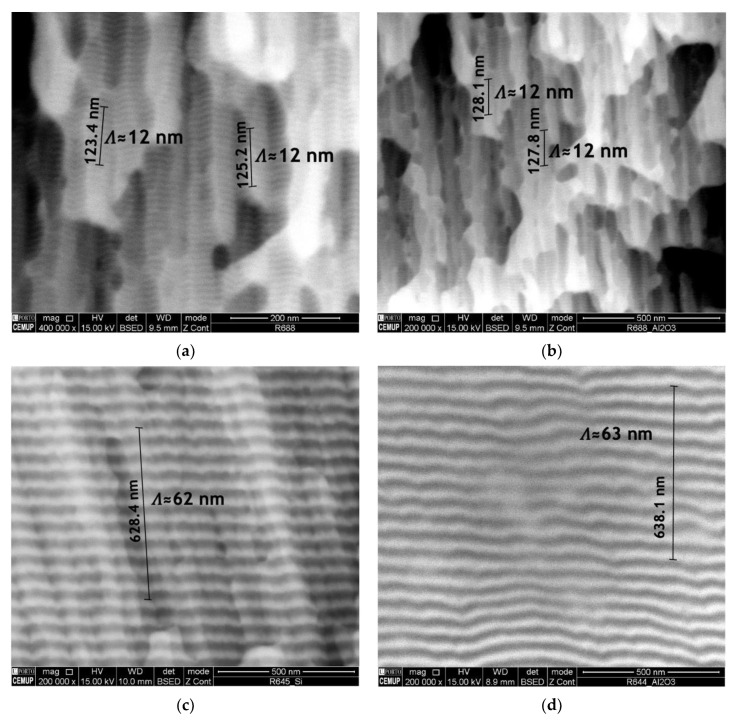
Electron backscattered (BSE) SEM images of Ni/Ti nanomultilayer thin films deposited onto (**a**) Si with Λ ≈ 12 nm, (**b**) Al_2_O_3_ with Λ ≈ 12 nm, (**c**) Si with Λ ≈ 60 nm and (**d**) Al_2_O_3_ with Λ ≈ 60 nm.

Ni/Ti nanomultilayer thin films with different modulation periods were deposited onto Ti6Al4V and Al_2_O_3_ substrates. The deposition onto silicon substrates allowed the modulation periods and average chemical composition to be obtained. The modulation period was estimated by dividing the total thickness by the product between deposition time and substrates’ rotation speed. Later, the period was checked by SEM. Short period Ni/Ti films were deposited onto Si at high rotation speeds to evaluate the average chemical composition by SEM/EDS and confirm that it is close to the equiatomic one. After achieving the desired average chemical composition, the depositions were carried out maintaining the power density applied to each target and varying the substrates’ rotation speed. The deposition of nanolayered films onto ceramics is a critical step due to the difficulty of good metallographic preparation of the surfaces and the low thermal conductivity. Figure 3 shows the cross-section of as-deposited nanomultilayers onto silicon and Al_2_O_3_ substrates. It can be seen that the nanolayered structure was preserved in both substrates, which means that no reactions occurred during the sputtering process. Another characteristic that can be observed is the typical columnar growth morphology, commonly reported in the literature for multilayer thin films deposited by magnetron sputtering [36,37,38]. Although in Figure 3a,b the nanolayers are barely distinguished using SEM, modulation periods near 12 nm can be measured for both substrates. In Figure 3c,d modulation periods slightly above 60 nm are observed, with Ti dark grey and Ni light grey layers clearly distinguishable.

### 3.2. Diffusion Bonding Using Ni/Ti Nanomultilayers

Microstructural and mechanical characterizations assessed the feasibility of joining Ti6Al4V to Al_2_O_3_ using Ni/Ti nanomultilayers with different modulation periods. The joints were processed at 750 and 800 °C, by applying a pressure of 5 MPa for 60 min.

**Figure 4:**
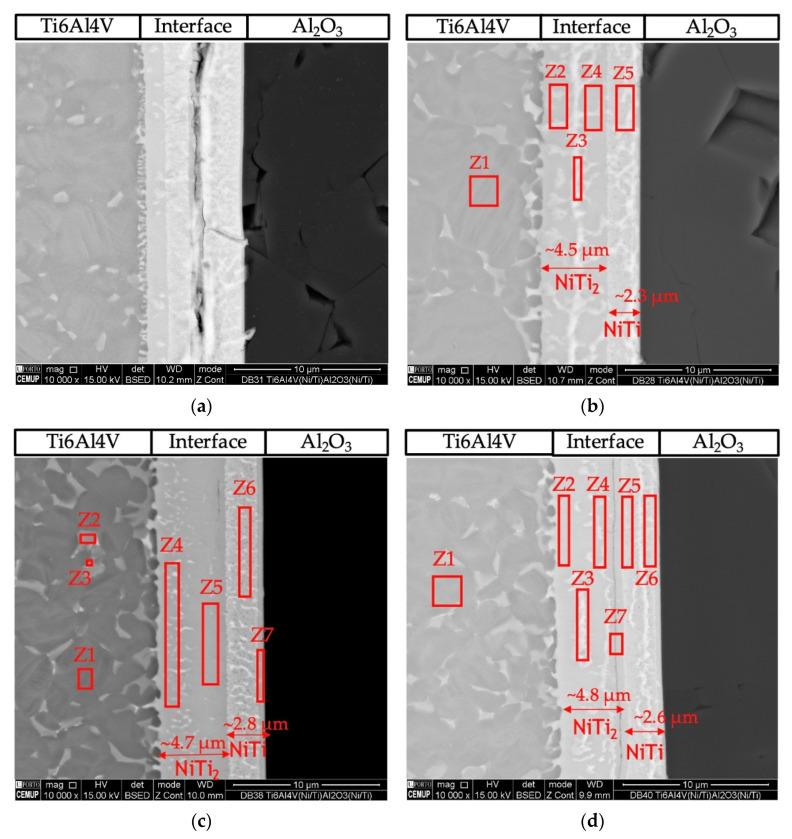

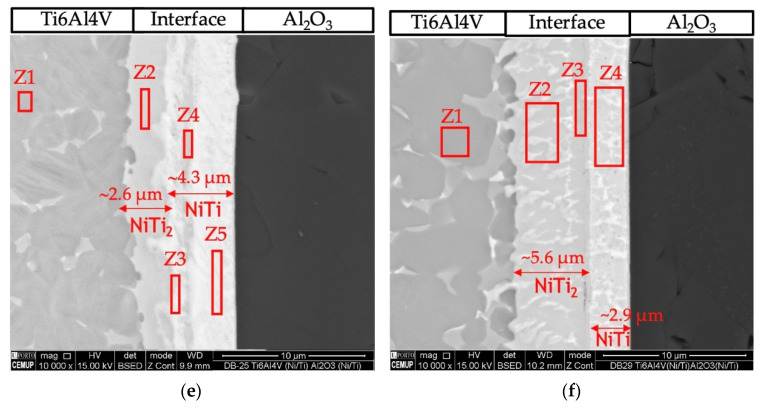
BSE SEM images of diffusion bonded Ti6Al4V/Al_2_O_3_ joints using Ni/Ti nanomultilayers processed using 5 MPa during 1 h at (**a**) 750 °C (Λ ≈ 12 nm), (**b**) 800 °C (Λ ≈ 12 nm), (**c**) 750 °C (Λ ≈ 25 nm), (**d**) 800 °C (Λ ≈ 25 nm), (**e**) 750 °C (Λ ≈ 60 nm), (**f**) 800 °C (Λ ≈ 60 nm).

Figure 4 presents SEM images of the cross-section of the joints obtained by solid-state diffusion bonding using nanomultilayers with three different modulation periods (Λ~12, 25 and 60 nm). A thin thickness characterizes the joints’ interfaces and no microstructural change or plastic deformation of the titanium alloy was observed when using an optimized pressure of 5 MPa. The modulation period seems to influence the success of bonding, as well as the bonding temperature. The number of layers observed at the interface and their morphology also depend on the period and bonding temperature. SEM images revealed interfaces with apparent soundness, except the joint processed at 750 °C using ~12 nm period nanomultilayers. In this case, unbonded areas and cracks are observed throughout the joint interface (Figure 4a). Although the 12 nm period is the closest to the peak reactivity of Ni/Ti multilayers, periods ≥ 25 nm seem more promising for assisting the diffusion bonding process of dissimilar materials. This result corroborates previous authors’ works on the diffusion bonding of metallic materials using nanoscale metallic multilayer thin films [38].

With the increase of the modulation period, at 750 °C, a few defects are observed at the interface (Figure 4c,e). For higher bonding temperature, fewer layers are observed at the interface, and a greater extent of diffusion of the interface elements towards Ti6Al4V is observed. At 750 °C, the interfaces obtained with the three modulation periods can be divided into three layers: layer 1 close to the Al_2_O_3_, layer 2 at the middle of the interface, and layer 3 close to the Ti6Al4V. As the temperature increases to 800 °C, the difference is the observation of only two layers at the interface, one on the Al_2_O_3_ side and a thicker one on the Ti6Al4V side.

**Figure 5:**
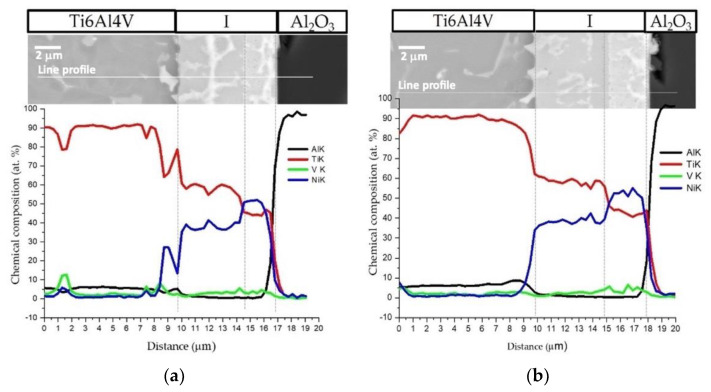
SEM image and EDS profiles across the joints processed at 800 °C/5 MPa/1 h using Ni/Ti nanomultilayers with (**a**) Λ ≈ 12 nm and (**b**) Λ ≈ 60 nm.

**Figure 6:**
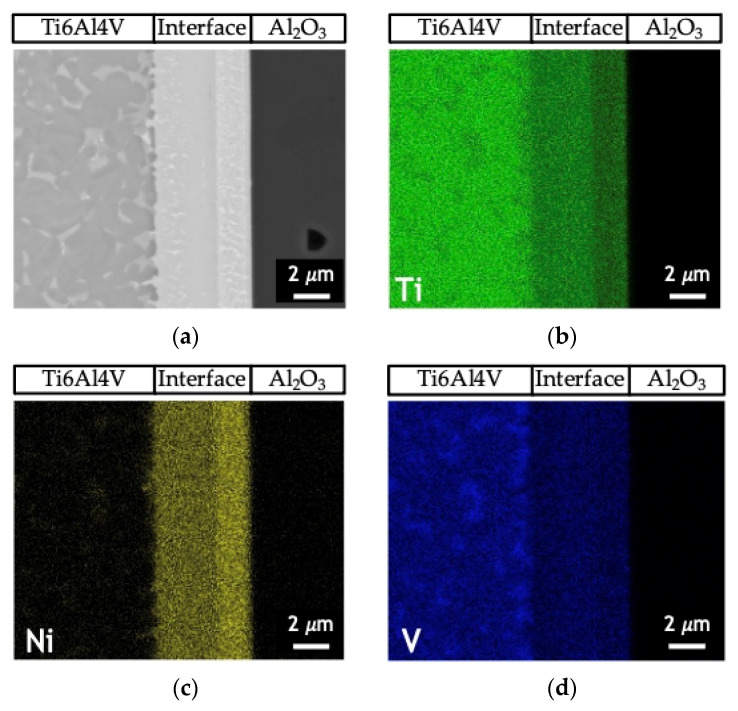
(**a**) SEM image and EDS elemental maps of (**b**) Ti (**c**) Ni and (**d**) V of the joint process at 750 °C/5 MPa/1 h using a Ni/Ti nanomultilayer with Λ ≈ 25 nm.

Figure 5 shows the EDS profiles for the joints processed at 800 °C using ~12 and 60 nm period Ni/Ti nanomultilayers. Based on EDS profiles, it is clear that the interfaces are mainly composed of Ti and Ni elements. A brighter phase with a higher amount of V is also identified due to the presence of this element in the as-deposited nanomultilayer thin films. Zones located close to Ti6Al4V present Ni-rich zones, confirming the diffusion of this element towards Ti6Al4V, increasing the β-Ti phase close to the interface since Ni is a β-stabilizer element. Similar results were obtained in diffusion bonding assisted with Ni/Ti multilayers [23,27]. The Ni and Ti diffusivities in Ti6Al4V and NiTi at 800 °C are significantly different which promotes a net diffusion of Ni from the interface towards the Ti6Al4V and Ti from the Ti6Al4V to the interface [39]. The diffusion of Ni from the interface towards Ti6Al4V, due to its high diffusivity, gives rise to a high amount of this element in the β-Ti phase. As the bonding temperature increases, an intense diffusion of Ni is promoted, resulting in a greater amount of β-Ti close to the interface. The EDS maps in Figure 6 clearly show that this interface is characterized by a Ni-rich layer near Al_2_O_3_ and a Ti-rich layer close to Ti6Al4V.

To identify the possible phases constituting the reaction layers formed at the interfaces, EDS chemical analyses were performed in conjunction with SEM observations and combined with the information provided by the Ni-Ti [40] and Ti-Al-Ni [40] phase diagrams. Table 1 presents the EDS results of each selected zone marked in the SEM images of Figure 4.

The joint interface processed at 800 °C using the Ni/Ti film with ~12 nm modulation period (Figure 3b) exhibits a continuous interface with a thickness similar to that obtained at 750 °C, but no cracks were detected. The interface consisted of two different layers. The layer adjacent to alumina (area marked as Z5) has a thickness of ~2.3 μm, and its chemical composition consists mainly of Ti (50.3 at.%) and Ni (45 at.%), which suggests that the NiTi phase was formed. On the opposite side, adjacent to Ti6Al4V (areas marked as Z2, Z3, and Z4), the main elements have average values of 62.3 ± 1.9 at.% Ti and 33.1 ± 2.8 at.% Ni, indicating that the NiTi_2_ phase is present.

Diffusion bonding using nanomultilayers with Λ ≈ 25 nm (Figure 3c,d) was successful at 750 and 800 °C. Both joints present interfaces with similar chemical composition and thickness. The EDS results suggest the phase NiTi_2_ was formed adjacent to Ti6Al4V, and on the oppositive side, close to Al_2_O_3_, the NiTi phase was identified. However, for the joint processed at 750 °C, a thin layer (Figure 4c, zone Z7) with a higher amount of Al (19.6 at.%) is present. However, a part of this Al can be attributed to the interaction volume with the alumina substrate. In Figure 4d the darker line indicated as Z7 can be regarded as a Ti-rich bond line, as already observed in previous works [26,27,41].

Figure 4e,f show images of the interfaces processed using Ni/Ti films with Λ ≈ 60 nm at 750 and 800 °C, respectively. The interfaces present chemical compositions similar to the other interfaces and it is possible to detect NiTi_2_ and NiTi layers. Figure 4f shows the interface of the joint obtained at 800 °C, and according to the EDS chemical compositions, a NiTi_2_ phase adjacent to Ti6Al4V is identified, while a NiTi phase formed adjacent to Al_2_O_3_.

**Figure 7:**
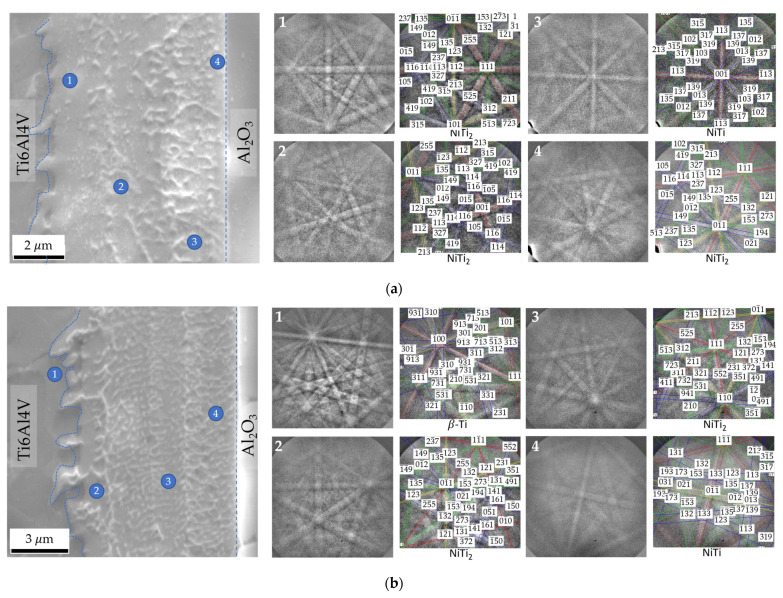
SEM images and EBSD Kikuchi patterns indexed as NiTi, NiTi_2_ and β-Ti phases of zones marked on the joints processed at 800 °C/5 MPa/1 h using Ni/Ti nanomultilayers with (**a**) Λ ≈ 12 nm and (**b**) Λ ≈ 60 nm.

The EBSD technique was used to confirm the phases at the interface; this technique allows Kikuchi patterns to be obtained in thin zones with a small interaction volume. Figure 7 displays EBSD Kikuchi patterns obtained for joints processed at 800 °C for 60 min using Ni/Ti films with ~12 and 60 nm periods. These results confirm the presence of the NiTi_2_ phase adjacent to Ti6Al4V. On the opposite side, close to Al_2_O_3_, the formation of the NiTi phase is identified. The joint produced with Λ ≈ 12 nm exhibits a thin layer close to the Al_2_O_3_ which has been identified as NiTi_2_ (Figure 7a). This layer was not identified by EDS due to its small size. As the period increases, this layer is not observed. The formation of layers composed of NiTi_2_ aligned grains has already been reported in previous works [27,36,41] when Ni/Ti multilayer thin films were used to assist the diffusion bonding of titanium alloys. The formation of the NiTi_2_ grains can be explained due to the decrease of the solubility of Ti in NiTi with the cooling from the bonding temperature. It can be also confirmed with this EBSD analysis that the β-Ti phase increases near the interface with the Ti6Al4V alloy due to the Ni diffusion, in accordance with the study of Cavaleiro et al. [42].

The microstructural characterization of the joints is in agreement with studies carried out on the microstructural evolution of Ni/Ti multilayer thin films with nanometric periods. In-situ thermal evolution studies [34,37,42] indicated that the first crystalline phase formed is NiTi (reacted multilayer), at temperatures of 375–400 °C, for Ni/Ti multilayers with different modulation periods, as a result of the Ni + Ti exothermic reaction. The formation of the NiTi_2_ phase at the interface has been reported in dissimilar diffusion bonding using Ni/Ti multilayer thin films when at least one of the base materials was the Ti6Al4V alloy [27,34,38,43]. Due to the diffusion of Ti from the Ti6Al4V alloy towards the interface and of Ni in the opposite direction already mentioned, a Ti enrichment is observed which promotes the formation of the NiTi_2_ layer close to this base material. Ni diffusion towards the Ti6Al4V also promotes the increase of the β-Ti phase close to the interface because Ni is a β-stabilizer element.

In previous authors’ work [23], without interlayer material, unsuccessful joining between Al_2_O_3_ and Ti6Al4V was reported at 800 °C during 60 min under 50 MPa of pressure. The joints produced using Ni/Ti nanomultilayers improved the contact surface, the diffusivity through the interface, and reduced residual stress allowing the success of the joining process at 800 °C/5 MPa/60 min, as well as at 750 °C/5 MPa/60 min. During the diffusion bonding process, Ni/Ti nanomultilayers acted as a localized heat source, releasing heat to the system and allowing the bonding conditions to be reduced, namely the temperature and pressure [26,27,30,41,43].

**Figure 8:**
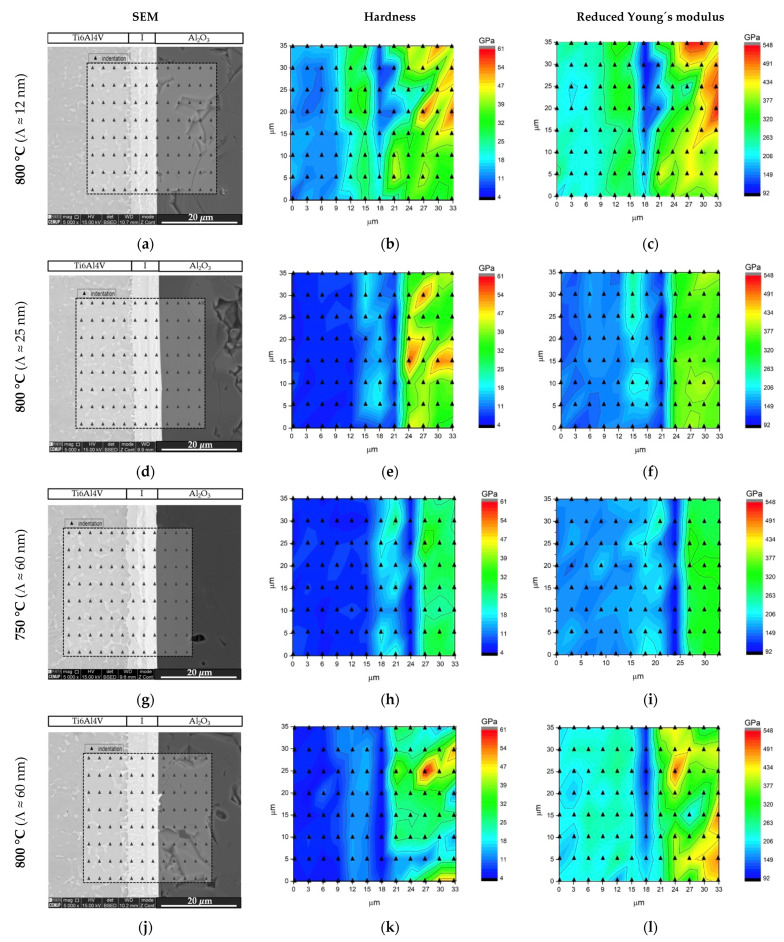
SEM images with the nanoindentation matrices marked, and hardness and reduced Young´s modulus (Er) maps across the joints processed using Ni/Ti nanomultilayers at (**a**–**c**) 800 °C (Λ ≈ 12 nm), (**d**–**f**) 800 °C (Λ ≈ 25 nm), (**g**–**i**) 750 °C (Λ ≈ 60 nm) and (**j**–**l**) 800 °C (Λ ≈ 60 nm).

Nanoindentation experiments were performed across the interface and zones adjacent to the base materials in order to obtain the joints’ hardness and reduce Young´s modulus (Er) distribution maps. Figure 8 shows hardness and Er maps for the joints processed at 800 °C using nanomultilayers with three different modulation periods, and for the joint processed at 750 °C using the highest modulation period Ni/Ti thin film.

The hardness maps show that the interface has a hardness value closer to Ti6Al4V, although a hardness increase is observed in the regions adjacent to the Ti alloy, identified as NiTi_2_. The variations of the hardness and Er values obtained by nanoindentation are expected due to the different phases that compose the interface. The distribution maps make it possible to clearly distinguish the NiTi and NiTi_2_ layers present at the interfaces. A lower Young’s modulus at the interface was expected near the Ti6Al4V side, since according to Young’s modulus values reported in the literature [44,45] E_NiTi2_ < E_NiTi_, E_Ti6Al4V_. Nevertheless, higher Er values were obtained in the layer identified as NiTi_2_, in accordance with previous dissimilar diffusion bonding experiments using Ni/Ti reactive nanomultilayers [23,34,43]. As the temperature increases, the hardness of the interface becomes more homogeneous (Figure 8h,k). This is related to the fact that increasing the bonding temperature results in more diffusion, which promotes homogeneity of the microstructure and chemical composition of the interface. Regarding the modulation period, it is noted that there is a decrease in the interface hardness with its increase. This may be related to the fact that the shorter the multilayer period, the smaller the grain size of the phases that form upon reaction during the joining process, as already reported in previous works [36,38]; as a result interfaces with slightly different hardness values are obtained.

The use of nanomultilayers as interlayer material proved to be a suitable approach that allows the joining of Ti6Al4V to Al_2_O_3_ without plastic deformation of the titanium alloy. The multilayers’ period has an influence on the microstructure of the interface and on the mechanical properties of the joints obtained. As the period decreases, the grain size also decreases, resulting in joints with higher hardness at the interface, which can be detrimental to the mechanical behavior. In addition, the hardness and reduced Young modulus maps revealed more homogeneous interfaces when using Ni/Ti nanomultilayers with Λ ≥ 25 nm, which confirms that these periods are more promising for joining purposes.

## 4. Conclusions

Diffusion bonding of Ti6Al4V to Al_2_O_3_ was processed at 750 and 800 °C, dwell time of 60 min and under an optimized pressure of 5 MPa using magnetron sputtered Ni/Ti multilayers with nanometric periods. The use of these nanomultilayers proves to be an effective approach since, without an interlayer, it was not possible to obtain sound joints between these base materials under the selected conditions.

The modulation period and the bonding temperature influence the number of layers formed at the joint interface, and as a consequence, the hardness and reduced Young’s modulus distribution maps. These nanoindentation maps highlight the different mechanical properties of the phases present across the joint interfaces. According to the microstructural analysis and mechanical behavior of the joints, the optimum multilayer periods are between 25 and 60 nm.

The joints’ interfaces are mainly composed of NiTi and NiTi_2_ phases. The layers adjacent to Ti6Al4V were identified as NiTi_2_ phase, while NiTi is identified in the layer adjacent to Al_2_O_3_. The intense interdiffusion of Ti and Ni between the Ti6Al4V and the interlayer material induces the formation of a higher amount of β-Ti phase close to the interface of this base material.

The use of the Ni/Ti nanomultilayers in the diffusion bonding of Ti6Al4V to Al_2_O_3_ presents a clear advantage in relation to the process without an interlayer material. The nanomultilayers improve the contact between the joining surfaces and enhance the joining between alumina and titanium alloy due to the improved reactivity and diffusivity resulting from their nanometric character.

## Figures and Tables

**Table 1:** Chemical composition (at.%) obtained by EDS of the zones marked in Figure 4.

Temperature/ Modulation Period Λ	Zone	Element (at.%)				Possible Phases
		Ti	Al	V	Ni	
800 °C/12 nm (Figure 4b)	1	88.6	9.7	1.7	-	α-Ti
	2	65.0	3.5	2.3	29.2	NiTi_2_
	3	60.8	1.5	2.6	35.1	NiTi_2_
	4	61.0	1.0	3.0	35.0	NiTi_2_
	5	50.3	1.4	3.3	45.0	NiTi
750 °C/25 nm (Figure 4c)	1	88.4	9.9	1.7	-	α-Ti
	2	74.9	4.6	14.0	6.5	β-Ti + NiTi_2_
	3	76.4	6.2	7.4	10.0	β-Ti + NiTi_2_
	4	64.0	3.8	2.2	30.0	NiTi_2_
	5	63.0	1.0	2.9	33.1	NiTi_2_
	6	50.5	0.5	3.6	45.4	NiTi
	7	48.5	19.6	2.6	29.3	α_2_-Ti_3_Al +AlNi_2_Ti + NiTi_2_
800 °C/25 nm (Figure 4d)	1	86.0	11.0	3.0	-	α-Ti
	2	65.0	4.4	1.8	28.8	NiTi_2_
	3	63.3	2.8	2.4	31.5	NiTi_2_
	4	65.0	1.8	3.0	30.2	NiTi_2_
	5	54.0	1.0	4.0	41.0	NiTi_2_+ NiTi
	6	50.7	1.5	3.8	44.0	NiTi
	7	68.0		3.0	29.0	NiTi_2_
750 °C/60 nm (Figure 4e)	1	88.1	10.1	1.8	-	α-Ti
	2	63.7	2.9	1.7	31.7	NiTi_2_
	3	50.5	0.6	3.4	45.5	NiTi
	4	53.0	0.6	3.8	43.6	NiTi+ NiTi_2_
	5	45.4	0.7	3.3	50.6	NiTi
800 °C/60 nm (Figure 4f)	1	87.0	11.0	2.0	-	α-Ti
	2	63.0	3.0	2.0	32.0	NiTi_2_
	3	60.0	0.9	3.0	36.1	NiTi_2_
	4	48.0	3.3	3.5	45.2	NiTi

## Data Availability

Not applicable.

## References

[1] Mir F.A., Khan N.Z., Parvez S. (2021). Recent advances and development in joining ceramics to metals. Mater. Today Proc..

[2] Simões S. (2018). Recent Progress in the Joining of Titanium Alloys to Ceramics. Metals.

[3] Cazajus V., Seguy S., Welemane H., Karama M. (2012). Residual stresses in a ceramic-metal composite. Appl. Mech. Mater..

[4] Leyens C., Peters M. (2003). Titanium and Titanium Alloys.

[5] Carter C.B., Norton M.G. (2007). Ceramic Materials.

[6] Zhang Y., Chen Y.-K., Yu D.-S., Sun D.-Q., Li H.-M. (2020). A review paper on effect of the welding process of ceramics and metals. J. Mater. Res. Technol..

[7] Uday M.B., Ahmad-Fauzi M.N., Noor A.M., Rajoo S., Ishak M. (2016). Current Issues and Problems in the Joining of Ceramic to Metal. Joining Technologies.

[8] Cai X.Q., Wang D.P., Wang Y., Yang Z.W. (2020). Microstructural evolution and mechanical properties of TiB2-TiC-SiC ceramics joint brazed using Ti-Ni composite foils. J. Eur. Ceram. Soc..

[9] Lu Y., Zhu M., Zhang Q., Hu T., Wang J., Zheng K. (2020). Microstructure evolution and bonding strength of the Al_2_O_3_/Al_2_O_3_ interface brazed via Ni-Ti intermetallic phases. J. Eur. Ceram. Soc..

[10] Li C., Zhang K., Mao X., Si X., Lan B., Liu Z.-G., Huang Y., Qi J., Feng J., Cao J. (2020). Microstructure and mechanical properties of the AlON / Ti6Al4V active element brazing joint. Mater. Sci. Eng. A.

[11] Emadinia O., Guedes A., Tavares C.J., Simões S. (2020). Joining Alumina to Titanium Alloys Using Ag-Cu Sputter-Coated Ti Brazing Filler. Materials.

[12] Cao J., Zheng Z.J., Wu L.Z., Qi J.L., Wang Z.P., Feng J.C. (2012). Processing, microstructure and mechanical properties of vacuum-brazed Al_2_O_3_/Ti6Al4V joints. Mater. Sci. Eng. A.

[13] Akselsen O.M. (1992). Diffusion bonding of ceramics. J. Mater. Sci..

[14] Travessa D., Ferrante M. (2002). The Al_2_O_3_-titanium adhesion in the view of the diffusion bonding process. J. Mater. Sci..

[15] Liu H.J., Feng J.C., Nogi K. (2004). Growth kinetics of reaction layers formed during diffusion bonding of SiC ceramic to TiAl alloy. Mater. Sci. Technol..

[16] Liao K.-H., Su C.-Y., Yu M.-Y. (2021). Interfacial microstructure and mechanical properties of diffusion-bonded W–10Cu composite/AlN ceramic using Ni–P and Ti interlayers. J. Alloys Compd..

[17] Liu J., Cao J., Song X., Wang Y., Feng J. (2014). Evaluation on diffusion bonded joints of TiAl alloy to Ti3SiC2 ceramic with and without Ni interlayer: Interfacial microstructure and mechanical properties. Mater. Des..

[18] Barrena M.I., Matesanz L., de Salazar J.M.G. (2009). Al_2_O_3_/Ti6Al4V diffusion bonding joints using Ag-Cu interlayer. Mater. Charact..

[19] Halbig M.C., Asthana R., Singh M. (2015). Diffusion bonding of SiC fiber-bonded ceramics using Ti/Mo and Ti/Cu interlayers. Ceram. Int..

[20] Yi J.L., Zhang Y.P., Wang X.X., Dong C., Hu H. (2016). Characterization of Al/Ti Nano Multilayer as a Jointing Material at the Interface between Cu and Al_2_O_3_. Mater. Trans..

[21] Xue H., Wei X., Guo W., Zhang X. (2020). Bonding mechanism study of active Ti element and α-Al_2_O_3_ by using first-principle calculation. J. Alloys Compd..

[22] Wang G., Sun X.N., Xu J.H., Shan Y., Han X., Xu J., Li J. (2019). Pressureless thermal diffusion bonding of transparent AlON ceramics by using a powder interlayer of parent material. Scr. Mater..

[23] Silva M., Ramos A.S., Vieira M.T., Simões S. (2021). Diffusion Bonding of Ti6Al4V to Al_2_O_3_ Using Ni/Ti Reactive Multilayers. Metals.

[24] Silva M., Ramos A.S., Simões S. (2021). Joining Ti6Al4V to alumina by diffusion bonding using titanium interlayers. Metals.

[25] Duarte L.I., Ramos A.S., Vieira M.F., Viana F., Vieira M.T., Koçak M. (2006). Solid-state diffusion bonding of gamma-TiAl alloys using Ti/Al thin films as interlayer. Intermetallics.

[26] Simões S., Viana F., Ramos A., Vieira M.T., Vieira M.F. (2018). Microstructural Characterization of Dissimilar Titanium Alloys Joints Using Ni/Al Nanolayers. Metals.

[27] Simões S., Viana F., Ramos A.S., Vieira M.T., Vieira M.F. (2013). Reaction zone formed during diffusion bonding of TiNi to Ti6Al4V using Ni/Ti nanolayers. J. Mater. Sci.

[28] Ma Y., Li H., Yang L., Hu A. (2018). Microstructures and reaction properties of Ti/Ni, Ti/Al and Ni/Al multilayer films. J. Nano Res..

[29] Zhu S., Włosiński W. (2001). Joining of AlN ceramic to metals using sputtered Al or Ti film. J. Mater. Process. Technol..

[30] Cao J., Song X.G., Wu L.Z., Qi J.J., Feng J.C. (2012). Characterization of Al/Ni multilayers and their application in diffusion bonding of TiAl to TiC cermet. Thin Solid Films.

[31] Adams D.P., Rodriguez M.A., McDonald J.P., Bai M.M., Jones E., Brewer L., Moore J.J. (2009). Reactive Ni/Ti nanolaminates. J. Appl. Phys..

[32] Ye J.-M., Lin Y.-P., Yang Y.-T., Chang J.-T., He J.-L. (2019). Electrochromic properties of Ni(V)Ox films deposited via reactive magnetron sputtering with a 8V–92Ni alloy target. Thin Solid Films.

[33] Yamamoto S., Yokomine T., Sato K., Terai T., Fukuda T., Kakeshita T. (2018). Ab Initio Prediction of Atomic Location of Third Elements in B2-Type TiNi. Mater. Trans..

[34] Cavaleiro A., Ramos A.S., Fernandes F., Schell N., Vieira M.T. (2020). Follow-up structural evolution of Ni/Ti reactive nano and microlayers during diffusion bonding of NiTi to Ti6Al4V in a synchrotron beamline. J. Mater. Process. Technol..

[35] Oliver W.C., Pharr G.M. (1992). An improved technique for determining hardness and elastic modulus using load and displacement sensing indentation experiments. J. Mater. Res..

[36] Simões S., Viana F., Ramos A.S., Vieira M.T., Vieira M.F. (2011). Anisothermal solid-state reaction of Ni/Al nanometric multilayers. Intermetallics.

[37] Cavaleiro A.J., Santos R.J., Ramos A.S., Vieira M.T.F. (2014). In-situ thermal evolution of Ni/Ti multilayer thin films. Intermetallics.

[38] Ramos A.S., Cavaleiro A.J., Vieira M.T., Morgiel J., Safran G. (2014). Thermal stability of nanoscale metallic multilayers. Thin Solid Films.

[39] He P., Liu D. (2006). Mechanism of forming interfacial intermetallic compounds at interface for solid state diffusion bonding of dissimilar materials. Mater. Sci. Eng. A.

[40] Zeng K., Schmid-Fetzer R., Huneau B., Rogl P., Bauer J. (1999). The ternary system Al–Ni–Ti Part II: Thermodynamic assessment and experimental investigation of polythermal phase equilibria. Intermetallics.

[41] Simões S., Ramos A.S., Viana F., Vieira M.T., Vieira M.F. (2017). TiAl diffusion bonding using Ni/Ti multilayers. Weld World.

[42] Cavaleiro A., Ramos A.S., Fernandes F., Baehts C., Vieira M.T. (2018). Interaction between Ni/Ti Nanomultilayers and Bulk Ti-6Al-4V during Heat Treatment. Metals.

[43] Simões S., Viana F., Ramos A.S., Vieira M.T., Vieira M.F. (2016). Microstructural characterization of diffusion bonds assisted by Ni/Ti nanolayers. J. Mater. Eng. Perform..

[44] Kipp D.O. Material Data Sheets. MatWeb, LLC, 2010; Online Version. http://www.matweb.com.

[45] Toprek D., Belosevic-Cavor J., Koteski V. (2015). Ab initio studies of the structural, elastic, electronic and thermal properties of NiTi_2_ intermetallic. J. Phys. Chem. Solids.

